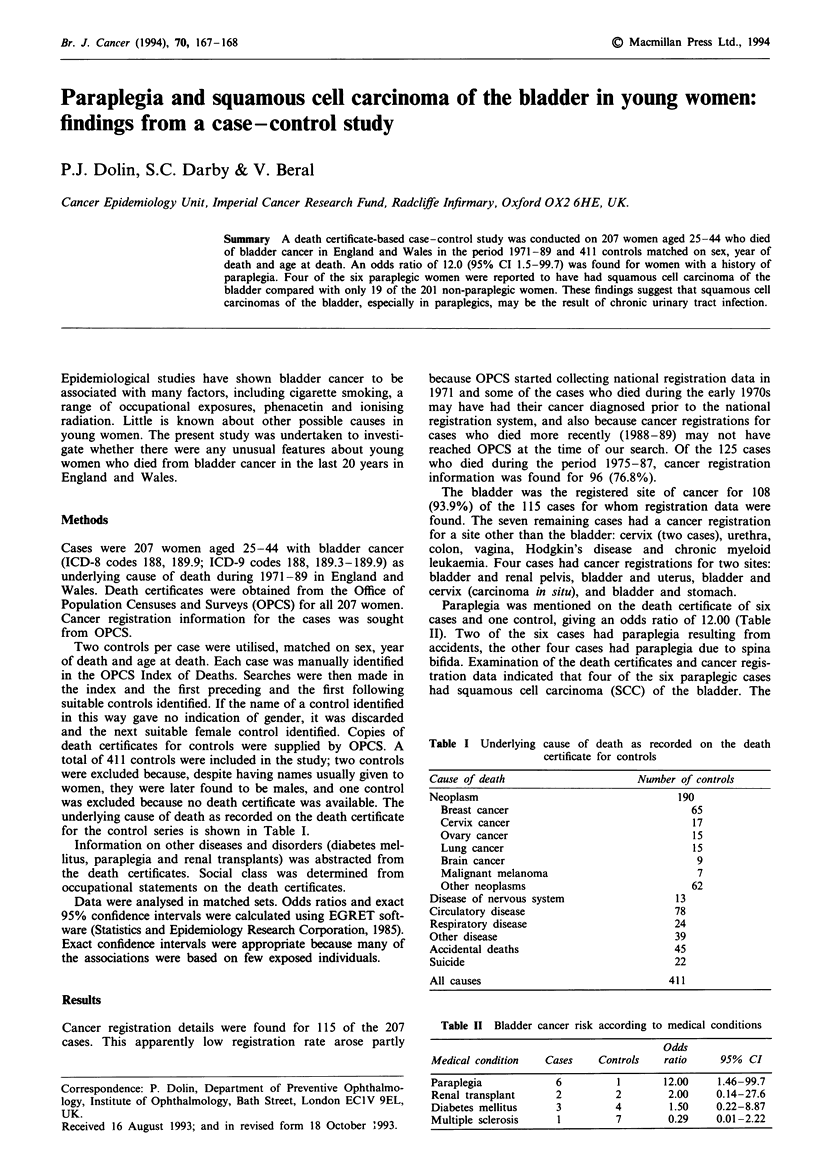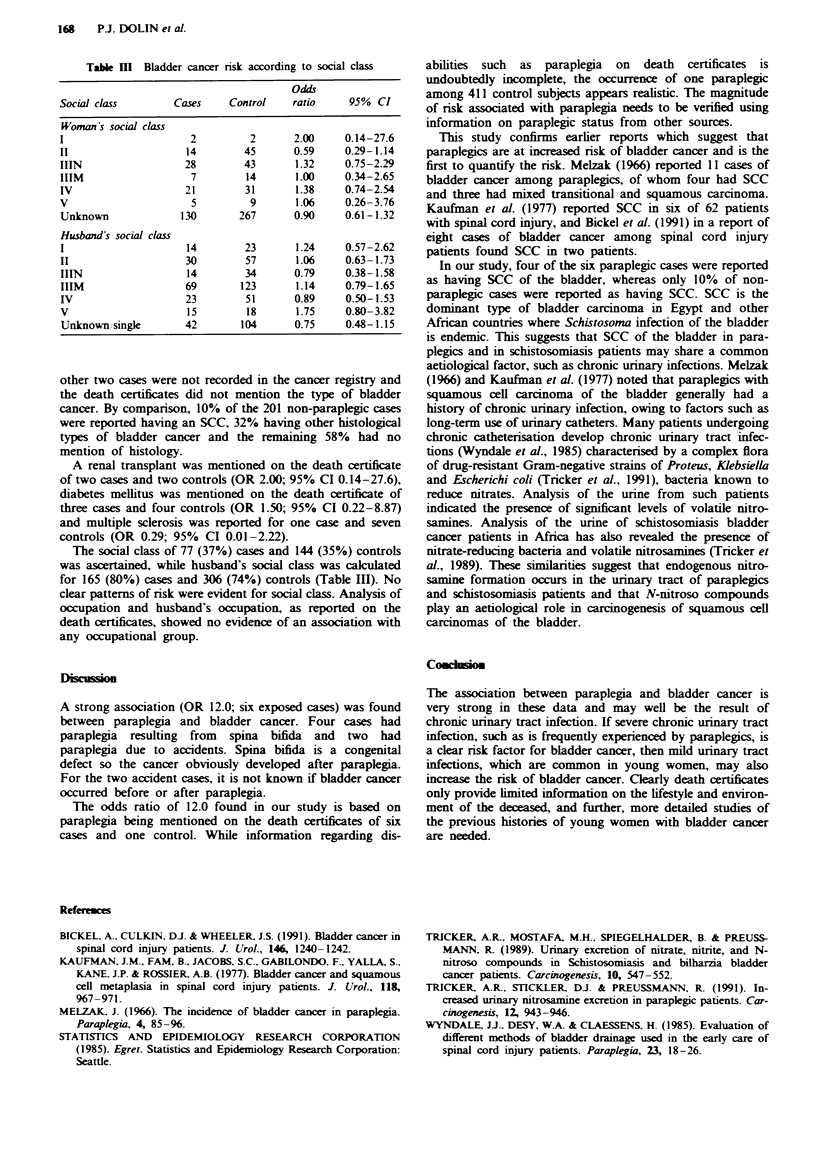# Paraplegia and squamous cell carcinoma of the bladder in young women: findings from a case-control study.

**DOI:** 10.1038/bjc.1994.269

**Published:** 1994-07

**Authors:** P. J. Dolin, S. C. Darby, V. Beral

**Affiliations:** Cancer Epidemiology Unit, Imperial Cancer Research Fund, Radcliffe Infirmary, Oxford, UK.

## Abstract

A death certificate-based case-control study was conducted on 207 women aged 25-44 who died of bladder cancer in England and Wales in the period 1971-89 and 411 controls matched on sex, year of death and age at death. An odds ratio of 12.0 (95% CI 1.5-99.7) was found for women with a history of paraplegia. Four of the six paraplegic women were reported to have had squamous cell carcinoma of the bladder compared with only 19 of the 201 non-paraplegic women. These findings suggest that squamous cell carcinomas of the bladder, especially in paraplegics, may be the result of chronic urinary tract infection.


Br. J. Cancer (1994), 70, 167–168 © Macmillan Press Ltd., 1994

### Paraplegia and squamous cell carcinoma of the bladder in young women: findings from a case–control study

P.J. Dolin, S.C. Darby & V. Beral

*Cancer Epidemiology Unit, Imperial Cancer Research Fund, Radcliffe Infirmary, Oxford OX2 6HE, UK.*

**Summary** A death certificate-based case–control study was conducted on 207 women aged 25–44 who died of bladder cancer in England and Wales in the period 1971–89 and 411 controls matched on sex, year of death and age at death. An odds ratio of 12.0 (95% CI 1.5–99.7) was found for women with a history of paraplegia. Four of the six paraplegic women were reported to have had squamous cell carcinoma of the bladder compared with only 19 of the 201 non-paraplegic women. These findings suggest that squamous cell carcinomas of the bladder, especially in paraplegics, may be the result of chronic urinary tract infection.

Epidemiological studies have shown bladder cancer to be associated with many factors, including cigarette smoking, a range of occupational exposures, phenacetin and ionising radiation. Little is known about other possible causes in young women. The present study was undertaken to investigate whether there were any unusual features about young women who died from bladder cancer in the last 20 years in England and Wales.

### Methods

Cases were 207 women aged 25–44 with bladder cancer (ICD-8 codes 188, 189.9; ICD-9 codes 188, 189.3–189.9) as underlying cause of death during 1971–89 in England and Wales. Death certificates were obtained from the Office of Population Censuses and Surveys (OPCS) for all 207 women. Cancer registration information for the cases was sought from OPCS.

Two controls per case were utilised, matched on sex, year of death and age at death. Each case was manually identified in the OPCS Index of Deaths. Searches were then made in the index and the first preceding and the first following suitable controls identified. If the name of a control identified in this way gave no indication of gender, it was discarded and the next suitable female control identified. Copies of death certificates for controls were supplied by OPCS. A total of 411 controls were included in the study; two controls were excluded because, despite having names usually given to women, they were later found to be males, and one control was excluded because no death certificate was available. The underlying cause of death as recorded on the death certificate for the control series is shown in Table I.

Information on other diseases and disorders (diabetes mellitus, paraplegia and renal transplants) was abstracted from the death certificates. Social class was determined from occupational statements on the death certificates.

Data were analysed in matched sets. Odds ratios and exact 95% confidence intervals were calculated using EGRET software (Statistics and Epidemiology Research Corporation, 1985). Exact confidence intervals were appropriate because many of the associations were based on few exposed individuals.

### Results

Cancer registration details were found for 115 of the 207 cases. This apparently low registration rate arose partly because OPCS started collecting national registration data in 1971 and some of the cases who died during the early 1970s may have had their cancer diagnosed prior to the national registration system, and also because cancer registrations for cases who died more recently (1988–89) may not have reached OPCS at the time of our search. Of the 125 cases who died during the period 1975–87, cancer registration information was found for 96 (76.8%).

The bladder was the registered site of cancer for 108 (93.9%) of the 115 cases for whom registration data were found. The seven remaining cases had a cancer registration for a site other than the bladder: cervix (two cases), urethra, colon, vagina, Hodgkin's disease and chronic myeloid leukaemia. Four cases had cancer registrations for two sites: bladder and renal pelvis, bladder and uterus, bladder and cervix (carcinoma *in situ*), and bladder and stomach.

Paraplegia was mentioned on the death certificate of six cases and one control, giving an odds ratio of 12.00 (Table II). Two of the six cases had paraplegia resulting from accidents, the other four cases had paraplegia due to spina bifida. Examination of the death certificates and cancer registration data indicated that four of the six paraplegic cases had squamous cell carcinoma (SCC) of the bladder. The

**Table I** Underlying cause of death as recorded on the death certificate for controls

| *Cause of death* | *Number of controls* |
|---|---|
| Neoplasm | 190 |
| Breast cancer | 65 |
| Cervix cancer | 17 |
| Ovary cancer | 15 |
| Lung cancer | 15 |
| Brain cancer | 9 |
| Malignant melanoma | 7 |
| Other neoplasms | 62 |
| Disease of nervous system | 13 |
| Circulatory disease | 78 |
| Respiratory disease | 24 |
| Other disease | 39 |
| Accidental deaths | 45 |
| Suicide | 22 |
| All causes | 411 |

**Table II** Bladder cancer risk according to medical conditions

| *Medical condition* | *Cases* | *Controls* | *Odds ratio* | *95% CI* |
|---|---|---|---|---|
| Paraplegia | 6 | 1 | 12.00 | 1.46–99.7 |
| Renal transplant | 2 | 2 | 2.00 | 0.14–27.6 |
| Diabetes mellitus | 3 | 4 | 1.50 | 0.22–8.87 |
| Multiple sclerosis | 1 | 7 | 0.29 | 0.01–2.22 |

Correspondence: P. Dolin, Department of Preventive Ophthalmology, Institute of Ophthalmology, Bath Street, London EC1V 9EL, UK.
Received 16 August 1993; and in revised form 18 October 1993.

Table III Bladder cancer risk according to social class

| Social class | Cases | Control | Odds ratio | 95% CI |
|---|---|---|---|---|
| *Woman's social class* | | | | |
| I | 2 | 2 | 2.00 | 0.14–27.6 |
| II | 14 | 45 | 0.59 | 0.29–1.14 |
| IIIN | 28 | 43 | 1.32 | 0.75–2.29 |
| IIIM | 7 | 14 | 1.00 | 0.34–2.65 |
| IV | 21 | 31 | 1.38 | 0.74–2.54 |
| V | 5 | 9 | 1.06 | 0.26–3.76 |
| Unknown | 130 | 267 | 0.90 | 0.61–1.32 |
| *Husband's social class* | | | | |
| I | 14 | 23 | 1.24 | 0.57–2.62 |
| II | 30 | 57 | 1.06 | 0.63–1.73 |
| IIIN | 14 | 34 | 0.79 | 0.38–1.58 |
| IIIM | 69 | 123 | 1.14 | 0.79–1.65 |
| IV | 23 | 51 | 0.89 | 0.50–1.53 |
| V | 15 | 18 | 1.75 | 0.80–3.82 |
| Unknown/single | 42 | 104 | 0.75 | 0.48–1.15 |

other two cases were not recorded in the cancer registry and the death certificates did not mention the type of bladder cancer. By comparison, 10% of the 201 non-paraplegic cases were reported having an SCC, 32% having other histological types of bladder cancer and the remaining 58% had no mention of histology.

A renal transplant was mentioned on the death certificate of two cases and two controls (OR 2.00; 95% CI 0.14–27.6), diabetes mellitus was mentioned on the death certificate of three cases and four controls (OR 1.50; 95% CI 0.22–8.87) and multiple sclerosis was reported for one case and seven controls (OR 0.29; 95% CI 0.01–2.22).

The social class of 77 (37%) cases and 144 (35%) controls was ascertained, while husband's social class was calculated for 165 (80%) cases and 306 (74%) controls (Table III). No clear patterns of risk were evident for social class. Analysis of occupation and husband's occupation, as reported on the death certificates, showed no evidence of an association with any occupational group.

### Discussion

A strong association (OR 12.0; six exposed cases) was found between paraplegia and bladder cancer. Four cases had paraplegia resulting from spina bifida and two had paraplegia due to accidents. Spina bifida is a congenital defect so the cancer obviously developed after paraplegia. For the two accident cases, it is not known if bladder cancer occurred before or after paraplegia.

The odds ratio of 12.0 found in our study is based on paraplegia being mentioned on the death certificates of six cases and one control. While information regarding disabilities such as paraplegia on death certificates is undoubtedly incomplete, the occurrence of one paraplegic among 411 control subjects appears realistic. The magnitude of risk associated with paraplegia needs to be verified using information on paraplegic status from other sources.

This study confirms earlier reports which suggest that paraplegics are at increased risk of bladder cancer and is the first to quantify the risk. Melzak (1966) reported 11 cases of bladder cancer among paraplegics, of whom four had SCC and three had mixed transitional and squamous carcinoma. Kaufman *et al.* (1977) reported SCC in six of 62 patients with spinal cord injury, and Bickel *et al.* (1991) in a report of eight cases of bladder cancer among spinal cord injury patients found SCC in two patients.

In our study, four of the six paraplegic cases were reported as having SCC of the bladder, whereas only 10% of non-paraplegic cases were reported as having SCC. SCC is the dominant type of bladder carcinoma in Egypt and other African countries where *Schistosoma* infection of the bladder is endemic. This suggests that SCC of the bladder in paraplegics and in schistosomiasis patients may share a common aetiological factor, such as chronic urinary infections. Melzak (1966) and Kaufman *et al.* (1977) noted that paraplegics with squamous cell carcinoma of the bladder generally had a history of chronic urinary infection, owing to factors such as long-term use of urinary catheters. Many patients undergoing chronic catheterisation develop chronic urinary tract infections (Wyndale *et al.*, 1985) characterised by a complex flora of drug-resistant Gram-negative strains of *Proteus*, *Klebsiella* and *Escherichi coli* (Tricker *et al.*, 1991), bacteria known to reduce nitrates. Analysis of the urine from such patients indicated the presence of significant levels of volatile nitrosamines. Analysis of the urine of schistosomiasis bladder cancer patients in Africa has also revealed the presence of nitrate-reducing bacteria and volatile nitrosamines (Tricker *et al.*, 1989). These similarities suggest that endogenous nitrosamine formation occurs in the urinary tract of paraplegics and schistosomiasis patients and that *N*-nitroso compounds play an aetiological role in carcinogenesis of squamous cell carcinomas of the bladder.

### Conclusion

The association between paraplegia and bladder cancer is very strong in these data and may well be the result of chronic urinary tract infection. If severe chronic urinary tract infection, such as is frequently experienced by paraplegics, is a clear risk factor for bladder cancer, then mild urinary tract infections, which are common in young women, may also increase the risk of bladder cancer. Clearly death certificates only provide limited information on the lifestyle and environment of the deceased, and further, more detailed studies of the previous histories of young women with bladder cancer are needed.

### References

BICKEL, A., CULKIN, D.J. & WHEELER, J.S. (1991). Bladder cancer in spinal cord injury patients. *J. Urol.*, **146**, 1240–1242.

KAUFMAN, J.M., FAM, B., JACOBS, S.C., GABILONDO, F., YALLA, S., KANE, J.P. & ROSSIER, A.B. (1977). Bladder cancer and squamous cell metaplasia in spinal cord injury patients. *J. Urol.*, **118**, 967–971.

MELZAK, J. (1966). The incidence of bladder cancer in paraplegia. *Paraplegia*, **4**, 85–96.

STATISTICS AND EPIDEMIOLOGY RESEARCH CORPORATION (1985). *Egret*. Statistics and Epidemiology Research Corporation: Seattle.

TRICKER, A.R., MOSTAFA, M.H., SPIEGELHALDER, B. & PREUSSMANN, R. (1989). Urinary excretion of nitrate, nitrite, and N-nitroso compounds in Schistosomiasis and bilharzia bladder cancer patients. *Carcinogenesis*, **10**, 547–552.

TRICKER, A.R., STICKLER, D.J. & PREUSSMANN, R. (1991). Increased urinary nitrosamine excretion in paraplegic patients. *Carcinogenesis*, **12**, 943–946.

WYNDALE, J.J., DESY, W.A. & CLAESSENS, H. (1985). Evaluation of different methods of bladder drainage used in the early care of spinal cord injury patients. *Paraplegia*, **23**, 18–26.